# Advance directives and end-of-life care preferences among adults in Wuhan, China: a cross-sectional study

**DOI:** 10.1186/s12889-021-12046-3

**Published:** 2021-11-08

**Authors:** Ping Ni, Bei Wu, Huijing Lin, Jing Mao

**Affiliations:** 1 School of Nursing, Tongji Medical College, Huazhong University of Science and Technology, number 13, Hangkong Road, Qiaokou District, Wuhan, Hubei China; 2 Rory Meyers College of Nursing and NYU Aging Incubator, New York University, New York, NY 10010 USA

**Keywords:** End-of-life, Advance directives, Chinese

## Abstract

**Background:**

Little is known about advance directives (ADs) and end-of-life (EOL) care preferences among the general population in Mainland China. This study aimed to describe knowledge and attitudes of ADs and EOL care preferences, and to explore factors related to preferences for ADs among Chinese adults.

**Methods:**

The sample included 1114 adult participants in Wuhan, Mainland China. A brief message including the link to the online survey was sent to local residents who were registered at household registration management centers in Wuhan. The questionnaire included information regarding demographics, self-rated health, views on ADs and EOL care. Bivariate analyses and binary forward logistic regression were conducted to examine factors related to ADs preferences of Chinese adults.

**Results:**

The average age of the sample was 48.0 years and more than half of the sample was female. 81.8% had never heard of ADs, but 86.6% indicated that they might create one after learning what ADs were. 58% would choose hospice care if they were terminally ill whereas 48.7% of the participants wanted to die at home. 92.3% would want to know their diagnosis and prognosis if ill; however, if their family members were diagnosed with an incurable disease, 50.5% would not tell their ill family member the actual diagnosis and prognosis. Those who had heard of ADs (OR = 1.567, *p* < 0.001), earned an associate’s degree (OR = 2.448, *p* < 0.001) or a bachelor’s degree or higher (OR = 2.382, *p* < 0.001), and self-rated their health as very poor/poor (OR = 1.002, *p* = 0.001) were more likely to be willing to make an AD than their counterparts. However, those who were single (OR = 0.149, *p* < 0.001) or widowed /divorced/separated (OR = 0.405, *p* = 0.001) were less likely to be willing to make an AD than the married ones.

**Conclusions:**

Chinese adults showed positive attitudes towards ADs. There is an urgent need to promote more educational initiatives and raise awareness on the importance of ADs. It is important to develop more policies and legislation about ADs to improve the quality of EOL care in Mainland China.

## Background

Death is an inevitable part of life [1]. The quality of life at the end of life is important for everyone [2] thus, most need to plan in advance for a “good death” [1]. It is well-known that palliative care, which includes advance care planning (ACP), improve a patient’s end-of-life (EOL) care, quality of death, and decrease psychological distress [3]. However, in Mainland China, development of palliative care is progressing very slowly, and there is no case law regarding Advance Directives (ADs) [4]. In addition, cultural tradition challenges the implementation of ADs in Mainland China given that the discussion of death is generally avoided, because it is considered as disrespectful [1].

In recent years, scholars and policymakers have increasingly recognized that it is important to provide quality palliative care. The World Health Organization stressed in its illness trajectory framework (2007) that palliative care should be provided according to the needs of patients as their disease progresses, not just at the end of life [2]. However, as noted, the development of palliative care in Mainland China has lagged far behind than those in developed countries. Mainland China was ranked 71 in the latest Quality of Death Index by the Economist Intelligence Unit that surveyed quality of EOL care in 80 countries/regions [5]. China has the highest life expectancy in the world [6]. Giving the higher life expectancy in Mainland China, the pattern of utilizing EOL care will become increasingly important in the future.

ACP is now more important than ever due to the COVID-19 pandemic, since the large number of affected patients and the limited capacity of the health care system. COVID-19 has challenged global healthcare systems and many countries are making efforts to improve them in order to provide good quality of care to patients [7]. It is clearer than ever that ACP is crucial for the general population.

There are a few studies exploring EOL care preferences among nursing home residents, [4] cancer patients, [8] and patients with chronic disease in China [9]. None have addressed the knowledge and attitudes towards ADs and EOL care preferences among the general population in Mainland China. Better public awareness and openness to discuss death and EOL issues are necessary for the completion of ADs [10]. Therefore, it is important to understand knowledge as well as attitudes towards ADs and EOL care among the general population. This study aimed to examine the knowledge of ADs and EOL care preferences, as well as to identify factors related to ADs preferences among adults in Mainland China.

## Methods

### Study design and participants

This is a cross-sectional online survey conducted via a survey platform. The participants of the study were local residents of Wuhan. Inclusion criteria for this study were: Chinese residents age 18 years and older and living in Wuhan. We selected 8 of a total of 13 communities of Wuhan, including Jiangan, Jianghan, Qiaokou, Hanyang, Wuchang, Qingshan, Hongshan, and Dongxihu. First, the 8 household registration management centers provided a list of the registered households. Then all the families in the list were contacted to participate in this study if they were interested via telephone calls. Participants who were interested in this study completed the survey through a link in a short text message. A total of 1114 participants were included in the study.

Ethical approval of the research protocol was granted by the Institutional Review Board of Huazhong University of Science and Technology and a signed informed consent was obtained from each participant. This study was conducted between April 2019 to March 2020.

### Data collection

Online surveys are an important tool for collecting data in the COVID-19 era when face-to-face survey methods are not feasible [11]. Participants’ knowledge and preferences for ADs and EOL care were measured by the tool [12] initially designed for Chinese older adults. This tool has been used among nursing home residents in Hong Kong [12] and Wuhan [4]. The dependent variable ‘AD preference’ was measured by asking “If you have a chance to make an AD when it is legal, will you make one?” It was coded as (0 = reluctant/fairly reluctant, 1 = willing/fairly willing). We also obtained participants’ sociodemographic factors and health status.

### Data analyses

The Statistical Package for Social Sciences (SPSS) version 23.0 (International Business Machines Corporation, Armonk, NY, USA) was used to conduct the analysis. Sample characteristics were analyzed using descriptive statistics. Means and standard deviations (SD) and frequencies (percentages) were used for continuous and categorical variables, respectively. Bivariate analyses were used to examine the associations of sociodemographic characteristics, health conditions, and other factors with the preferences for AD. Significant variables from the bivariable analysis were entered into a forward logistic regression analysis. The significance level was .05 in this study.

## Results

### Characteristics of participants

The characteristics of the 1114 participants are presented in Table 1. Of the recruited participants, 65.3% were female. Almost a third (31.8%) of the participants’ educational level was high school or below, 28.8% had earned an associate’s degree, and more than one third (39.4%) had a bachelor degree and above. Most (90.3%) participants did not have religious beliefs. Most (84.6%) of the participants were married or in a cohabitation status, 80.3% of them owned a private house, and 93.2% had non-medical related jobs. Most (83.1%) of the participants rated their financial status as enough or AD more than enough. Over two-thirds (68.4%) had medical insurance, while 75.0% did not have social pension. Nearly half (53.4%) of the participants’ spouse/son/daughter/parents/others were the breadwinner. A quarter of the participants (25.3%) had a chronic disease, about 1.4% were terminally ill, and 59.4% self-rated their health as very bad/bad. More than half did not have family members with chronic disease (53.9%) and 85.1% of them did not have family members with a terminal disease (85.1%).

**Table 1:** Characteristics of Participants (*n* = 1114)

Sociodemographic factors	n (%)
Age^*^	
18–44	468 (42.0)
45–59	426 (38.2)
>60	220 (19.7)
sex	
male	387 (34.7)
female	727 (65.3)
education	
high school and below	354 (31.8)
associate’s degree	321 (28.8)
bachelor degree and above	439 (39.4)
religious belief	
No religious belief	1006 (90.3)
Catholic/Christian/Buddhist/Taoist	108 (9.7)
marital status	
single	77 (6.9)
widowed/divorced/separated	95 (8.5)
cohabitation/married	942 (84.6)
house type	
private house	895 (80.3)
public house/rented house/others	219 (19.7)
occupation	
medical related job	76 (6.8)
Non-medical related job	1038 (93.2)
Economic level	
can’t make ends meet/not enough	188 (16.9)
enough/more than enough	926 (83.1)
medical insurance	
No	352 (31.6)
Yes	762 (68.4)
Social pension	
No	836 (75.0)
Yes	278 (25.0)
main breadwinner of the family	
myself	519 (46.6)
spouse/son/daughter/parents/others	595 (53.4)
chronic disease	
yes	282 (25.3)
no	832 (74.7)
terminally ill	
yes	16 (1.4)
no	1098 (98.6)
self-rated health	
very good/good	452 (40.6)
very bad/bad	662 (59.4)
Be the main care giver of family members with chronic disease	
no family members with chronic disease	601 (53.9)
yes	513 (46.1)
be the main care giver of family members with terminal disease	
no family members with terminal disease	948 (85.1)
yes	166 (14.9)

*Mean: 48.03, SD 12.459

### Knowledge and preferences for ADs and end-of-life care

Table 2 shows that 81.8% have never heard of ADs before, but after learning what an AD was, 86.6% were willing/fairly willing to make one if it was legal. The main reason was “To ease the burden on my family to make decisions for myself”. While the main reason cited for reluctant/fairly reluctant to make an AD was “It is too early to make one”, followed by “The law is not perfect”, “It is no use to make one” and “Not familiar with it”.

**Table 2:** Knowledge of AD, AD and End-of-Life Preferences Among Chinese Adult Population (*n* = 1114)

Items	n (%)
Have you heard of AD^*^ before?	
Yes	203 (18.2)
No	911 (81.8)
If you have a chance to make an AD which is legal, will you make one?	
Willing/fairly willing	965 (86.6)
Reluctant/fairly reluctant	149 (13.4)
Reasons to make an AD (multiple choice questions)	
To ease the burden on my family to decide for me	691 (62.0)
It’s good to make my family members know my EOL care preferences in advance	593 (53.2)
It is good to arrange my future affairs in advance	575 (51.6)
Others	21 (1.9)
Reasons to reject AD (multiple choice questions)	
It is too early to make one	528 (47.4)
The law is not perfect	371 (33.3)
It is no use to make one	371 (33.3)
Not familiar with it	330 (29.6)
My family members will arrange all the affairs for me	206 (18.5)
My decision may change	165 (14.8)
It may bring bad luck if make one	83 (7.5)
Others	41 (3.7)
Have you heard of Life-sustaining treatment?	
No	414 (37.2)
Yes	700 (62.8)
If you were terminally ill, you will prefer …	
Only choose appropriate hospice care that can provide comfort, even though it can’t prolong life	643 (57.7)
Euthanasia	201 (18.0)
Give up treatment	176 (15.8)
To prolong life as much as possible even with severe pain or uncomfortable	52 (4.7)
Others	42 (3.8)
Who will be designated as your proxy decision maker if you become unconscious?	
Spouse	588 (52.8)
Son/daughter	298 (26.8)
Doctor in charge	112 (10.1)
Parents	68 (6.1)
Other family members	28 (2.5)
Other persons	17 (1.5)
Friends	3 (0.3)
Who should decide whether the patient should receive life-sustaining treatment?	
Patient, family member and doctor in charge decide together	482 (43.3)
Patient and doctor in charge decide together	154 (13.8)
Patient	143 (12.8)
Family member and doctor in charge decide together	98 (8.8)
Patient and family member decide together	81 (7.3)
Doctor in charge	80 (7.2)
Family member	76 (6.8)
Where do you want to live your rest life and die?	
My home	543 (48.7)
Hospice care center	415 (37.3)
Hospital	64 (5.7)
Other place	58 (5.2)
Nursing home	34 (3.1)
Would you still prefer to die at home if you can’t get enough support from family members or medical staff?	
Yes	232 (42.7)
No	311 (57.3)
If you were diagnosed with incurable disease, which kind of following things would be the most important for you to live your rest life? (multiple choices)	
Surrounded by spouse/relatives and friends	761 (68.3)
Not to be a burden to others	615 (55.2)
Having privacy and died with dignity	576 (51.7)
Be in familiar environment	516 (46.3)
Can be helped by medical professionals	414 (37.2)
Can be supported by professional medical staff in emergency	342 (30.7)
My own wishes can be heard and respected	329 (29.5)
Regional, spiritual and cultural requirements can be met	124 (11.1)
If you were diagnosed with incurable disease, would you want to know your diagnosis and prognosis?	
Yes	1028 (92.3)
No	86 (7.7)
If your family member were diagnosed with incurable disease, would you tell him/her the actual diagnosis and prognosis?	
Yes	551 (49.5)
No	563 (50.5)
Whom should be involved with end of life treatment and care discussion with medical staff if a patient was diagnosed with a terminal disease?	
Patient and his/her family member	734 (65.9)
Only patient himself/herself	295 (26.5)
Only patient’s family member	85 (7.6)

*AD: Advance Directives

Two-thirds of the sample (62.8%) had heard of life-sustaining treatments and 57.7% indicated that they would choose hospice care that can provide comfort, even though it can’t prolong life, if they were terminally ill. Over half (52.8%) would choose their spouses as their proxy decision maker if they became unconscious. 43.0% thought patients, family members, and doctors should decide together regarding whether the patient should receive life-sustaining treatments. For the place of death, 48.7% wanted to live their rest life and die at home, followed by the hospice care center (37.3%). If they can’t get enough support from family members or medical staff, 57.3% did not prefer to die at home.

When participants were asked what would be the most important factors of life if they were diagnosed with an incurable disease, the top five were: “Surrounded by spouse/relatives and friends”(68.3%), “Not to be a burden to others”(55.2%), “Having privacy and died with dignity”(51.7%), “Be in familiar environment”(46.3%), “Can be helped by medical professionals”(37.2%).

Regarding diagnosis and prognosis, the majority of participants (92.3%) wanted to know if they were diagnosed with incurable disease. If their family members were diagnosed with an incurable disease, 50.5% would not inform their ill family member of the actual diagnosis and prognosis. Two thirds (65.9%) thought both the patient and their family member should be involved with EOL treatment decision-making and discussion with medical staff.

### Factors related to the adult Population’s preference for an AD

Bivariate analyses identified sex, educational level, marital status, house type, social pension, the main breadwinner of the family, the main caregiver of the family member with chronic disease/terminal disease, whether heard of ADs, self-rated health, and economic level as statistically significant factors related to ADs preferences. In the logistic regression model, the following four factors were the significant factors associated with preference for making an AD: having an associate’s degree (odds ratio [OR] 2.448) or a bachelor’s degree and higher (OR 2.382), heard of ADs before (OR 1.567), self-rated health as very bad/bad (OR 1.002) and were single (OR 0.149) or widowed/divorced/separated (OR 0.405) (Table 3).

**Table 3:** Logistic Regression analysis of Independent Predictors of Preference to make an AD (*n* = 1114)

Independent Predictors (Willing vs Reluctant)	OR^†^	95%CI	*P*
**Educational Level**			<.001
High school level or lower (reference group)			
Associate’s degree	2.448	1.485–4.037	<.001
Bachelor degree and higher	2.382	1.495–3.795	<.001
Have heard of AD^*^?			
Yes	1.567	1.224–2.007	<.001
No (reference group)			
**Self-rated health condition**			
very bad/bad	1.002	1.001–1.003	0.001
very good/good (reference group)			
**Marital status**			<.001
cohabitation/married (reference group)			
single	0.149	0.083–0.267	<.001
widowed/divorced/separated	0.405	0.236–0.695	0.001

*AD: Advance Directives

OR^†^:Odds Ratio

## Discussion

### Preferences for an AD among adults

Similar to previous studies that were conducted among older adults or older adults with chronic diseases, [1, 2, 4, 8, 13, 14] only a very small portion of those in this sample in Wuhan, Mainland China have heard of ADs. Nonetheless, most of them indicated positive attitudes after learning about what comprises an AD. This finding is similar to the studies conducted in Hong Kong [15] and Macao [16]. The low awareness of ADs and the positive attitudes towards it suggests a need for enhancing the promotion of ADs and potentially considering legislation to support ADs [4] in Mainland China.

### End-of-life care preferences among the adult population in mainland China

Our study showed that more than half (57.7%) of the participants would prefer hospice care, focusing on comfort care, even though it cannot prolong life if they are terminally ill. This finding is lower than the study conducted (87.6%) in Hong Kong [2]. Chinese adults in Hong Kong have adopted ADs early, and the general population may be more familiar with hospice care due to the issue of “Substitute Decision Making and Advance Directives” which released in the year of 2002 [17]. In Mainland China, only one agency was established in 2013 to popularize ADs and enhance dying with dignity [18]. Thus, public knowledge about ADs in Hong Kong may be more widespread compared with the general population in Mainland China. Hospice care is getting increased attention in China. On February 9th, 2017 the National Health Commission of People’s Republic of China issued the Basic Standards, Management Regulations and Practice Guideline of Hospice Care Center (Trial version) of China in order to facilitate the development of hospice care [19]. On December 5th, 2019, 71 cities/districts in Mainland China were required to provide hospice care [19]. The policy may promote the awareness and acceptance of hospice care among the general population, which can explain the reason why hospice care centers were the second location choice (home was the first choice) that the general population preferred to die in. In our study, more than half would prefer not to die at home if they cannot get enough support from family members and medical staff. This emphasizes the importance of professional home-based hospice care support as well as the development of professional hospice care centers in Mainland China.

Similar to Ivo’s study, [20] spouses were nominated as the proxy decision maker by more than half of the participants once they became unconscious, followed by their sons or daughters. Nearly half of the participants reported that patients, family members and doctors should decide together regarding whether the patient should receive life-sustaining treatments. This was very different from a previous study [4] which found that nearly half of the Chinese nursing home residents chose their doctors to be the surrogate decision maker about life-sustaining treatments. In Chinese nursing homes, older residents believe their doctors are more qualified due to professional knowledge and expertises, [21] and physician-patient relationships have been traditionally rooted in paternalism [22]. Younger adults were more likely to choose collective decision making with the development of palliative care.

For place of death, our study found that most participants preferred to die at home, to be surrounded by spouse/relatives and friends, not to be a burden to others, and die with dignity in a familiar environment. This reinforces the need to develop home-based EOL care, and make sure that participants’ EOL care preferences are respected and documented in an AD [4]. For the diagnosis and prognosis notification, it is interesting that almost all the participants wanted to know their diagnosis and prognosis if they were diagnosed with an incurable disease. However, if their family members were diagnosed with an incurable disease, a little more than half of them would not inform their ill family member of the actual diagnosis and prognosis. It is because Chinese people are strongly influenced by Confucianism which stresses the virtues of mercy, love, and humanity [23]. On one hand, they want to know the diagnosis and prognosis to make themselves ready for illness and death, as they do not want to be a burden to their families [24]. On the other hand, they have the responsibility to take care of their family members [25]. The norm of filial piety may lead to adults’ withholding the diagnosis and prognosis from their family members [26, 27] to avoid potential adverse effects like emotional distress and pressure [28] of telling them the truth. But whether telling the truth would bring adverse effects needs to be further explored, as studies in China have yielded inconsistent results about the effects of disclosing a life-threatening diagnosis/prognosis to patients [27–29]. Some studies showed that Chinese are not opposed to discussing EOL issues [30]. These results reinforce the need to strengthen training for medical staff and family members so they can report patients of their diagnosis and prognosis skillfully. An example is to inform them in a more indirect manner, using discussions about examples of other persons’ circumstances or hypothetical scenarios to inform [31] them and learn more about their treatment preferences.

### Factors related to ADs preferences among adults in mainland China

Higher levels of education was found to be associated with making an AD, which was similar to other studies [13, 32]. Education was associated with health literacy [32] and high health literacy may facilitate the person to understand and make an AD [33]. Low educational levels and poor medical knowledge may prevent discussions about ADs [25]. In Mainland China, the knowledge level of ADs was low, thus more education programs need to be carried out to the general population to raise awareness of ADs and thus, the readiness to make an AD.

Similar to other studies [4, 13] that those who had heard of ADs were more willing to make an AD. Public education regarding the concept of ADs is needed to raise awareness and knowledge as providing ADs related information or explanation about ADs promotes ADs completion [33, 34].

Adults who self-rated their health as poor were more likely to make an AD than their counterparts. This finding was similar to a previous study which indicating that those with poor self-rated health levels were more likely to make an AD [32]. Higher awareness of their own vulnerability may motivate them to make an AD [32]. Other studies also found that a poor health condition or deteriorating health was associated with a higher tendency to make an AD [4, 30, 35]. These results suggested that discussions of EOL care preferences should be carried out early before adults’ health gets worse, so that they can have time to discuss their ADs preferences. This is particularly the case currently because of the COVID-19 pandemic, when the infected patients’ health condition deteriorates abruptly and unexpectedly.

Compared with the married adults, those who were single or widowed/divorced were less likely to make an AD. For married/cohabiting couples, they may have more supportive relationship with each other, which will increase the likelihood to name their spouse as their proxy decision maker [36]. Other studies also indicated that greater emotional support and better marital quality are positively associated with ACP [37–39]. For the unmarried adults, it is often uncertain who will be appointed as the proxy decision maker, as making an AD involves naming a health care proxy.

It is likely that general public’s’ attitudes towards ADs and ACP may have changed after the outbreak of COVID-19. The COVID-19 pandemic may have highlighted the importance of ADs and ACP due to limited medical resources, [40] and the public become more aware of the fact that health can deteriorate suddenly and unexpectedly [41]. Our study showed that only few adults had heard of ADs, but most of them had positive attitudes toward ADs after learning about it. COVID-19 may have provided important opportunities to promote ACP. Education interventions are needed to promote ACP at the post COVID-19 era in China.

## Limitations and strengths

There are several limitations in our study. First, it is a cross-sectional design. Second, there is sampling bias, because we only included those who registered in eight household registration management centers in Wuhan and who volunteered to participate in our study. Those who lived in Wuhan may be very different from their rural counterparts. The findings from this study cannot be generalizable to the entire population of China. Third, only those residents who owned smart cell phone could access the questionnaire interlink, and those who owned the old ordinary mobile phone, which are likely to be common among those with a lower level of socioeconomic status, may have different opinions about ADs from the general population. However, the number of people who do not own a smart cell phone is likely to be very low in Wuhan, China. Nonetheless, our study has some strengths. A large sample was used in this study, and this is the first study that has been conducted on knowledge about ADs and EOL care among Chinese adults.

## Conclusions and implications

In our study, those who were with higher education, who had heard of ADs, had a poor self-rated health, and were married or cohabitating, were more likely to make an AD. Although the majority of adults had never heard of ADs, most of them preferred to make an AD after learning what ADs were. This suggests that there are great opportunities to promote ADs among the general population in Mainland China. Moreover, the sample participants indicated a preference for hospice care, and to die at home with professional medical help or in a hospice care center. Professional home-based hospice care models and hospice care centers need to be established in Mainland China. The participants also preferred to know the diagnosis and prognosis if they get an incurable disease. However, if their family members were diagnosed with an incurable disease, half of them would not tell their family members the actual diagnosis. The medical staff and family members need to be trained to inform the patients of their diagnosis and prognosis skillfully. ADs need to be promoted in China to help adults’ EOL care wishes be fulfilled. Policy about ADs/ACP should be made to respect adults’ rights in Mainland China. Online educational materials and social media may be helpful in encouraging EOL discussions and promote ADs awareness under the conditions of the COVID-19 pandemic [40].

## Data Availability

The data used in this study are available and can be provided by the corresponding author on a reasonable request.

## Abbreviations

AD: Advance directives
EOL: end-of-life
ACP: Advance care planning
CLHLS: Chinese Longitudinal Healthy Longevity Survey
SPSS: Statistical Package for Social Sciences
OR: odds ratio
